# Effect of Resistance Training with Different Set Structures on Neurotrophic Factors and Obesity-Related Biomarkers in Middle-Aged Korean Women with Obesity

**DOI:** 10.3390/jcm12093135

**Published:** 2023-04-26

**Authors:** Hyun Seok Bang

**Affiliations:** Department of Sport Rehabilitation, Tong Myong University, Busan 48520, Republic of Korea; banghs@tu.ac.kr; Tel.: +82-51-629-2142

**Keywords:** set structure, muscle strength, brain-derived neurotrophic factor, nerve growth factor, adiponectin, leptin

## Abstract

This 12-week study investigates the effects of resistance training with different set structures on the plasma levels of brain-derived neurotrophic factor (BDNF), nerve growth factor (NGF), and obesity-related markers in middle-aged Korean women with obesity. A total of 40 middle-aged women with obesity (mean age, 59.87 ± 2.80 years) were enrolled in this study; only 31 women were able to complete the 12-week exercise period. The participants were randomly divided into the following four groups: control (CON, *n* = 8), drop set system (Drop, *n* = 8), descending set system (Descend, *n* = 7), and ascending set system (Ascend, *n* = 8). Body composition was recorded, and blood samples were obtained and evaluated before and after the 12-week exercise period intervention. Two groups showed no significant differences in body weight, body fat percentage, and body mass index before and after the 12-week exercise period. However, significant differences were observed in the blood levels of BDNF, NGF, adiponectin, leptin, and glucagon-like peptide-1 (GLP-1). BDNF and NGF showed significant differences in both time and interaction effects. Post hoc analysis revealed that the Drop group had higher BDNF and NFG levels than those of the Ascend and CON groups, while there was a significant increase in the levels of these biomarkers in the Descend and Drop groups in the time comparison. Adiponectin levels also showed significant differences in both time and interaction effects. Post hoc analysis revealed that the Drop and Descend groups had higher adiponectin levels than those of the CON group. Leptin levels decreased significantly in the Descend and Drop groups over time. GLP-1 levels showed no significant differences between the groups; however, there were significant differences in both time and interaction effects. Post hoc analysis revealed that the Drop group had lower GLP-1 levels than those of the CON group. This study suggests that resistance training with different set structures can have varying effects on the blood levels of different biomarkers in middle-aged women with obesity. These findings have implications for exercise prescription, and may provide insights into the mechanisms underlying the beneficial outcomes of resistance training in middle-aged Korean women with obesity.

## 1. Introduction

Resistance training is a popular form of exercise that has been extensively researched for its ability to improve the muscle strength, body composition, and overall health of individuals of all ages and genders [1,2]. However, despite studies on the numerous benefits of resistance training [3,4], there is limited research on its impact on neurotrophic factors and obesity-related markers in middle-aged women with obesity.

Middle-aged women with obesity are at a higher risk of developing metabolic disorders such as Type 2 diabetes, hypertension, and cardiovascular disease [5,6]. Obesity can lead to insulin resistance and inflammation that can damage to the blood vessels and other organs [7]. Additionally, middle-aged women are particularly susceptible to obesity due to their hormonal changes and reduced physical activity [8].

Brain-derived neurotrophic factor (BDNF) and nerve growth factor (NGF) are crucial molecules involved in neural growth, synaptic maturation, axonal growth, and synaptic plasticity [9]. These factors stimulate neural regeneration, and influence the development and plasticity of the nervous system [10]. Adiponectin, leptin, and glucagon-like peptide-1 (GLP-1) are important hormones that regulate the metabolism and energy balance, and their dysregulation is associated with obesity and related metabolic disorders [11,12,13].

Few studies have investigated the effects of resistance training with different set structures on BDNF, NGF, adiponectin, leptin, and GLP-1 blood levels in middle-aged women with obesity. Therefore, this study compares the effects of three different set structures of resistance training on blood levels of these biomarkers in middle-aged women with obesity. Understanding the impact of resistance training with different set structures on these outcomes may provide valuable insights into the design of effective exercise interventions for individuals with obesity.

In this study, we hypothesized that a resistance training program with a drop-set structure would result in greater improvements in BDNF, NGF, adiponectin, leptin, and GLP-1 levels compared to those with other set structures. The findings of this study may contribute to the development of effective exercise interventions to improve brain function and metabolic health in middle-aged Korean women with obesity.

## 2. Materials and Methods

### 2.1. Participants and Study Design

**Figure 1:**
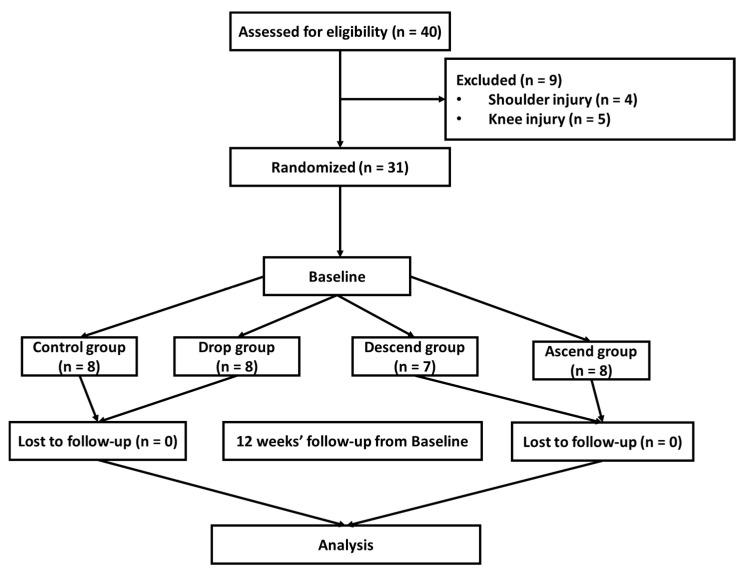
Flow diagram of the study.

A total of 40 middle-aged Korean women with obesity (mean age, 59.87 ± 2.80 years) agreed to participate in this study. However, only 31 participants were able to complete the 12-week exercise plan. The inclusion criteria were as follows: (1) women aged ≥ 56 years, (2) body mass index (BMI) ≥ 27, (3) fat % ≥ 36, (4) not exercising ≥ 1 once a week, and (5) no experience in resistance exercise. The exclusion criteria were as follows: (1) serious health issues, including cardiovascular disease, psychiatric disorders, and brain-related diseases; (2) any medication for hormones and metabolism; (3) habitual lifestyle problems, including smoking and alcoholism. Of the participants, 9 did not complete the 12-week exercise plan due to shoulder or knee injuries. The participants were randomly divided into the following four groups: control (CON, *n* = 8), drop set system (Drop, *n* = 8), descending set system (Descend, *n* = 7), and ascending set system (Ascend, *n* = 8). Body composition and blood samples were obtained and evaluated before and after the 12-week intervention. After 12 weeks, all parameters were determined at the same time in the morning after one day to remove the effect of acute resistance exercise and after 12 h of fasting. The resistance exercise groups were subjected to an exercise program (09:30 a.m., 5 days/week, and 60 min/day) that included warm-up (5 min), main exercise (45 min), and cool down (10 min) for 12 weeks. The control group did not participate in any exercise program, and performed their routine (normal) physical activities. All participants were requested to maintain their physical activity and dietary patterns during the intervention. All guidelines for the resistance exercise program was followed using standardized protocols by expert investigators. All participants provided their informed consent before participation. All study protocols adhered to the Declaration of Helsinki and were approved by the institutional review board (05/2021). The flowchart of the study is shown in Figure 1. The characteristics of the participants are presented in Table 1.

### 2.2. One Repetition Maximum (1RM)

After 3 days of practice in each session, the participants performed a maximal incremental test. The 1RM test was performed using the indirect measurement methods described in a previous study [14]. Briefly, the 1RM is defined as the maximal load that indicates the muscular strength of an individual.

### 2.3. Resistance Training Program

The resistance training program consisted of 8 major muscle group exercises: chest press, let pulldown, leg extension, leg curl, shoulder press, bicep curl, triceps extension, and abdominal crunch. Participants performed 3 sets of 8–12 repetitions at an intensity of 70–80% of their 1RM for each exercise with 1–2 min of rest between sets. The program lasted for 12 weeks, with participants performing the program for 60 min per day, 5 days per week. Participants were initially trained on the proper technique for each exercise, and supervised during the first few sessions to ensure proper form and progression. The program was progressed by increasing the weight by 5–10% once participants had been able to perform 12 repetitions with good form for all sets of an exercise. Exercise intensity was measured every 2 weeks, and graded exercise intensity was induced to correctly maximize muscle strength. The number of repetitions was controlled, with 8 repetitions per set for the first 1–4 weeks, 10 repetitions per set for weeks 5–8, and the number of repetitions was not controlled for weeks 9–12 and was until exhaustion. To maintain the same exercise intensity, participants were controlled and minimized on the basis of the rate of perceived exertion (RPE), and were instructed to avoid performing other exercises during the study. The exercise program was supervised by a specialized exercise expert, and the program is presented in Table 2 and Table 3.

### 2.4. Assessment of Body Composition

The participants rested for 1 h. After rest, body composition was measured using bioelectrical impedance analysis (BIA) (In-body 720, Biospace Co., Seoul, Korea). We measured the height (cm), body weight (kg), and body fat mass percentage (BFP) (%), and the BMI was calculated as the body mass divided by height squared (kg/m^2^), pre- and postintervention [15].

### 2.5. Blood Collection and Measurements

Venous blood was obtained from the participants pre- and postexercise intervention, following 12 h of fasting (09:00 a.m.). Blood was collected in plasma-separating tubes, centrifuged at 3000 rpm for 10 min to obtain the plasma, and subsequently stored at −80 °C until analysis. BDNF (R&D Systems, Minneapolis, MN, USA), NGF (R&D Systems, Minneapolis, MN, USA), adiponectin (R&D System, Minneapolis, MN, USA), leptin (R&D System, Minneapolis, MN, USA) and GLP-1 (Cusabio, Wuhan, China) blood levels were analyzed using enzyme-linked immunosorbent assay (ELISA) kits, following the manufacturer’s instructions. Absorbance was measured using a microplate spectrophotometer (SpectraMax M2e Microplate Reader; CA, USA).

**Table 3:** Resistance exercise program.

Items	Day	Program
Warm-up (15 min)		Working and stretching
Main exercise (25 min)	Monday	Seated chest press
		Lying dumbbell fly
		Cable cross over fly
		Pec deck fly machine
		Crunch
	Tuesday	Stated leg press
		Leg extension machine
		Leg curl machine
		Standing calf raise machine
		Reverse crunch
	Thursday	Chinning assistant machine
		Let pull down machine
		One arm dumbbell row
		Seated cable row machine
		Crunch
	Friday	Barbell curl machine
		Hammer curl
		Lying triceps barbell extension
		Cable triceps pull down
		Reverse crunch
	Saturday	Seated shoulder press machine
		Side lateral raise machine
		Dumbbell front raise
		Back lateral raise machine
		Crunch
Cooldown (20 min)	Data	Walking and stretching

### 2.6. Statistics

All data are presented as the mean ± standard deviation. Data analysis was performed using SPSS 26.0 (SPSS Inc., Chicago, IL, USA). A Shapiro–Wilk normality test was conducted for each variable on the basis of the pretest values to determine data normality [15]. Time point and repeated-measures ANOVA were conducted to test the mean differences. For the interaction effects, the significance of each group and time point was tested using syntex pairwise comparison. The significance level for all variables was set at *p* < 0.05.

## 3. Results

The baseline characteristics of the participants were similar among the four exercise groups, indicating successful randomization (Table 4). We assessed body weight, body mass index (BMI), and body fat percentage to investigate the effects of different set structures on body composition. However, our results show no statistically significant differences in body composition between the different set groups (*p* > 0.05). Thus, the current findings suggest that the 12-week exercise protocol did not lead to significant changes in the body composition of middle-aged Korean women with obesity.

The rationale for assessing BDNF and NGF levels was to investigate the effects of resistance training on neurotrophic factors in middle-aged women with obesity. The analysis of neurotrophic factors shows that BDNF levels had significant differences with respect to both time (*p* < 0.01) and interaction effects (*p* < 0.05). Post hoc analysis revealed that the Drop group had higher levels than those of the Ascending and Control groups, and there was a significant increase in the Descending and Drop groups in the time comparison. Similarly, NGF levels showed significant differences for both time (*p* < 0.001) and interaction effects (*p* < 0.01). Post hoc analysis revealed that the Drop group had higher levels than those of the Control group, and there was a significant increase in the Descending and Drop groups in the time comparison (Table 5). Thus, the current findings suggest that different set structures may have differential effects on neurotrophic factors in middle-aged Korean women with obesity.

We assessed adiponectin, leptin, and GLP-1 to investigate the effects of different set structures on obesity-related biomarker in middle-aged women with obesity. Adiponectin levels did not differ significantly between the groups; however, there were significant differences in the time (*p* < 0.001) and interaction (*p* < 0.05) effects. Post hoc analysis revealed that the Drop and Descending groups had higher adiponectin levels than those of the Control group. In the time comparison, there was a significant increase in all groups.

Leptin levels did not show significant differences in either group or interaction effects; however, there was a significant difference in time (*p* < 0.01). Post hoc analysis revealed a significant decrease in the Descending and Drop groups.

GLP-1 levels did not differ significantly between the groups; however, there were significant differences in both the time (*p* < 0.001) and interaction effects (*p* < 0.05). Post hoc analysis revealed that the Drop group had lower levels than those of the Control group. In the time comparison, there was a significant decrease in all groups. Thus, the current findings suggest that different set structures may contribute to improving obesity-related biomarkers in middle-aged Korean women with obesity (Table 6).

## 4. Discussion

To our knowledge, this is the first study to investigate the effects of a 12-week resistance exercise intervention with different set structures on the blood levels of BDNF, NGF, and obesity-related biomarkers in middle-aged Korean women with obesity. Our findings revealed no differences in body weight, BFP, or BMI, but the drop set group of participants showed the greatest improvement in BDNF and NGF levels. In addition, the drop and descending set groups showed the greatest improvement in adiponectin and GLP-1 levels.

Resistance exercise was an effective intervention for improving body composition by reducing body fat in middle-aged women with obesity [16,17]. However, the results of the present study showed no significant differences in body weight, BFP, or BMI among the groups. This is consistent with previous studies that failed to show significant changes in body composition after resistance exercise interventions in adults generally and healthy older adults [18,19]. This result suggests that participant characteristics such as genetics, age, baseline fitness levels, and exercise study design, including exercise parameters such as the type, time, intensity, and frequency of exercise, make it difficult to observe significant changes in body weight, BFP, and BMI after 12 weeks of resistance exercise.

BDNF and NGF are critical for neural growth, synaptic maturation, axonal growth, and synaptic plasticity [20]. These factors play an essential role in neural regeneration, and the development and plasticity of the nervous system [21]. In particular, resistance exercise stimulates neural regeneration and influence the development and plasticity of the nervous system. In particular, resistance exercise stimulates neural regeneration, and influences the development and plasticity of the nervous system. In this study, we investigated the effects of different set structures of resistance exercise on BDNF and NGF blood levels in middle-aged Korean women with obesity. Our results show that the drop-set resistance exercise regimen led to a significant increase in BDNF levels compared to the other groups. This finding is consistent with previous research indicating that resistance exercise can lead to an increase in BDNF and NGF levels [22]. Moreover, previous studies have shown that exercise can enhance learning and memory by increasing BDNF and NGF levels [23]. However, most of these studies focused on aerobic exercises and combined aerobic and resistance exercises [24]. Therefore, our study provides novel evidence that the drop-set regimen of resistance exercise may be a more effective training for strengthening cognitive function in middle-aged women with obesity. Our findings indicate that drop-set resistance exercise interventions can increase NGF and BDNF levels in middle-aged Korean women with obesity, which may have significant clinical implications for enhancing cognitive function and mood regulation. Our study adds to the growing body of literature that highlights the potential neuroprotective effects of resistance exercise interventions in individuals with neurological disorders. It is worth noting that while our study supports the idea that resistance exercise can increase BDNF and NGF levels, our sample was small. Future studies should evaluate intermediate processes, such as after 4, 8, and 16 weeks, and compare the results with those of other studies to justify the duration of the study.

Adiponectin is an adipokine that plays crucial roles in regulating glucose and lipid metabolism, insulin sensitivity, and inflammation. The present study investigated the effects of resistance training with different set structures on blood adiponectin levels in middle-aged Korean women with obesity. The results showed no significant difference in adiponectin levels between the groups; however, there were significant differences in both time and interaction effects. These findings are consistent with other studies that reported a positive effect of resistance exercise training on adiponectin levels [25]. In contrast, Ward et al. (2020) demonstrated that resistance exercise resulted in significantly decreased adiponectin levels in middle-aged women [26]. Although recent studies have debated the effect of resistance exercise on adiponectin levels, this discrepancy may be due to the different resistance exercise protocols employed. Furthermore, other studies have suggested that resistance exercise training is a promising strategy for improving adiponectin levels and reducing the risk of metabolic disorders in this population.

Leptin is a hormone that plays an important role in energy balance regulation [27,28]. Some studies investigated changes in leptin levels after exercise [29], but not after resistance exercise with different set structures. This study investigated the effect of resistance exercise with different set structures on leptin levels. In our study, there were no significant differences in either group or interaction effects; however, there was a significant difference in time. To our knowledge, this is the first study to suggest that there were no significant changes between the groups. Similarly, previous studies suggested that resistance exercise decreases leptin levels [30,31]. 

GLP-1 is a hormone secreted by the intestine in response to food intake and plays a crucial role in regulating blood glucose metabolism [32,33]. In the present study, we found no significant differences in GLP-1 levels in the various exercise groups, but there were significant differences in both time and interaction effects. Post hoc analysis revealed that the Drop group had lower GLP-1 levels than those of the Control group. Furthermore, in the time comparison, there was a significant decrease in GLP-1 levels in all groups (Ascending, Descending, and Drop groups). Previous studies suggested that resistance exercise decreases GLP-1 levels, resulting in improved cognitive function [34]. Our present findings suggest that resistance exercise may be an effective intervention for reducing metabolic disorders in middle-aged Korean women with obesity.

The present study has several limitations. First, the sample was small (*n* < 8 in each group). Second, this study evaluated only a subset of women with obesity, namely, middle-aged women with obesity. Third, the present study did not measure blood sugar levels, hemoglobin A1C levels, diet records, and muscular strength pre- and postintervention. Lastly, we did not measure the sustainability of changes in the biomarker levels after cessation of the intervention. Future studies need a larger number of participants of both healthy women and those with obesity, and to collect more comprehensive data such as blood sugar levels, hemoglobin A1C levels, diet records, and muscular strength measurements.

## 5. Conclusions

We investigated the effects of a 12-week resistance exercise program with different set structures on the blood levels of BDNF, NGF, and obesity-related markers in middle-aged women with obesity. The results revealed no differences in body weight, BFP, and BMI, but the drop set group showed the greatest improvement in BDNF and NGF levels. In addition, the drop and descending set groups showed the greatest improvement in adiponectin and GLP-1 levels. These findings may contribute to the development of resistance training programs to improve health problems in middle-aged women with obesity.

## Figures and Tables

**Table 1:** Characteristics of participants.

Groups ^1^	Years	Hight (cm)	Weight (kg)	BMI (kg/m^2^)	Fat (%)
CON	60.62 ± 2.77	157.37 ± 6.32	66.62 ± 5.47	27.38 ± 1.90	36.87 ± 4.61
Drop	60.00 ± 2.97	158.37 ± 5.42	70.25 ± 5.23	27.98 ± 0.81	38.12 ± 3.64
Descend	59.42 ± 2.57	156.85 ± 4.81	68.42 ± 4.79	27.82 ± 1.74	37.85 ± 2.96
Ascend	59.37 ± 3.20	155.00 ± 5.20	66.62 ± 4.43	27.78 ± 2.17	38.12 ± 3.39

^1^ Control group (CON, *n* = 8); drop set system group (Drop, *n* = 8); descending set system group (Descend, *n* = 7); ascending set system group (Ascend, *n* = 8).

**Table 2:** Resistance exercise intensity.

Variables	Drop	Descend	Ascend
1 set	40%	60%	40%
2 set	55%	55%	50%
3 set	65%	50%	55%
4 set	45%	40%	60%
Total	205%	205%	20%

Drop set system group (Drop, *n* = 8), descending set system group (Descend, *n* = 7), and ascending set system group (Ascend, *n* = 8).

**Table 4:** Body composition of participants before and after resistance training.

Variables	CON		Drop		Descend		Ascend		Source	*F*	Post-Hoc
	Before	After	Before	After	Before	After	Before	After			
Weight (kg)	67.75 ± 5.06	67.75 ± 3.91	70.25 ± 5.23	69.12 ± 3.52	68.42 ± 4.79	67.57 ± 4.23	66.62 ± 4.43	66.25 ± 3.88	T	3.095	NS
									G	0.571	
									T × G	0.639	
Fat (%)	36.87 ± 4.61	36.62 ± 4.17	36.12 ± 3.64	35.37 ± 2.68	35.85 ± 2.96	34.71 ± 2.42	38.12 ± 3.39	37.62 ± 2.50	T	2.478	NS
									G	0.142	
									T × G	1.889	
BMI (kg/m^2^)	27.38 ± 1.91	27.40 ± 1.83	27.98 ± 1.84	27.56 ± 1.76	27.82 ± 1.74	27.51 ± 2.26	27.77 ± 2.17	27.61 ± 1.93	T	2.571	NS
									G	1.101	
									T × G	0.671	

Control group (CON, *n* = 8), drop set system group (Drop, *n* = 8), descending set system group (Descend, *n* = 7), and ascending set system group (Ascend, *n* = 8), T: time, G: group, *F*: ANOVA *F*-value represents the significance level of the analysis of variance test conducted for the comparison of the mean values among the groups, NS: no significance.

**Table 5:** Neural-related biomarkers of participants before and after resistance training.

Variables	CON		Drop		Descend		Ascend		Source	*F*	Post-Hoc
	Before	After	Before	After	Before	After	Before	After			
BDNF (pg/dL)	179.87 ± 29.21	178.37 ± 17.33	181.12 ± 31.05	227.02 ± 24.35	176.42 ± 17.58	207.71 ± 34.13	171.01 ± 26.15	176.42 ± 17.58	T	14.640 **	C > A,D
									G	2.986	
									T × G	3.027 *	
NGF (pg/dL)	35.62 ± 3.73	36.25 ± 6.49	32.25 ± 5.44	47.12 ± 11.28	37.00 ± 5.97	43.85 ± 5.89	35.62 ± 7.57	38.12 ± 7.377	T	17.436 ***	C > D
									G	1.065	
									T × G	4.692 **	

Control group (CON, *n* = 8), drop set system group (Drop, *n* = 8), descending set system group (Descend, *n* = 7), and ascending set system group (Ascend, *n* = 8), T: time, G: group, F: (ANOVA F-value) represents the significance level of the analysis of variance test conducted for the comparison of the mean values among the groups, A: ascending set system group, B: descending set system group, C: drop set system group. D: control group, * *p* < 0.05, ** *p* < 0.01, *** *p* < 0.001.

**Table 6:** Obesity-related biomarkers of participants before and after resistance training.

Variables	CON		Drop		Descend		Ascend		Source	*F*	Post-Hoc
	Before	After	Before	After	Before	After	Before	After			
Adiponectin (ng/mL)	279.50 ± 27.74	272.62 ± 36.47	291.37 ± 26.10	311.12 ± 24.70	287.28 ± 28.83	308.71 ± 38.68	286.12 ± 26.90	305.50 ± 32.13	T	7.033 ***	B,C > D
									G	1.211	
									T × G	4.435 *	
Leptin (pg/mL)	164.62 ± 24.85	166.12 ± 26.81	159.75 ± 20.35	141.62 ± 16.70	167.75 ± 28.23	152.75 ± 15.38	162.07 ± 18.85	152.75 ± 31.81	T	11.699 **	NS
									G	2.107	
									T × G	0.611	
GLP-1 (ng/mL)	1.72 ± 0.51	1.77 ± 0.50	1.68 ± 0.50	1.34 ± 0.34	1.74 ± 0.45	1.37 ± 0.30	1.86 ± 0.44	1.53 ± 0.43	T	18.256 ***	C > D
									G	0.558	
									T × G	3.031 *	

Control group (CON, *n* = 8), drop set system group (Drop, *n* = 8), descending set system group (Descend, *n* = 7), and ascending set system group (Ascend, *n* = 8), T: time, G: group, F: ANOVA F-value represents the significance level of the analysis of variance test conducted for the comparison of the mean values among the groups, NS: none significant, A: ascending set system group, B: descending set system group, C: drop set system group. D: control group, * *p* < 0.05, ** *p* < 0.01, *** *p* < 0.001.

## Data Availability

The data presented in this study can be obtained by contacting the corresponding author.
